# Isolation of Thermophilic Bacteria *Geobacillus subterraneus* From Mount Tangkuban Perahu and the Novelty as a Candidate for *Streptococcus mutans* Anti-Biofilm

**DOI:** 10.1155/ijod/4285984

**Published:** 2024-11-25

**Authors:** Emma Rachmawati, Shinta Asarina, Gabriel Bagus Kennardi, Akeyla Tabina Tawangalun, Candra Arumimaniyah, Kartika Indah Sari, Hening Tjaturina Pramesti, Ratu Safitri, Ani Melani Maskoen

**Affiliations:** ^1^Department of Oral Biology, Faculty of Dentistry, Universitas Padjadjaran, Bandung, Indonesia; ^2^Microbiology Laboratory Assistant, Faculty of Medicine, Universitas Padjadjaran, Bandung, Indonesia; ^3^Biotechnology Student, Postgraduate School, Bandung Technology Institute, Bandung, Indonesia; ^4^Department of Biology, Faculty of Mathematics and Natural Sciences, Universitas Padjadjaran, Bandung, Indonesia; ^5^Senior Lecturer of the Biotechnology Study Program, Graduate School, Universitas Padjadjaran, Bandung, Indonesia

**Keywords:** antimicrobial peptides (AMPs), biofilm, *Geobacillus subterraneus*, *Streptococcus mutans*, thermophilic bacteria

## Abstract

Thermophilic bacteria living in extreme areas with high temperatures are capable of producing secondary metabolites, such as antimicrobial peptides (AMPs). AMPs are stable at high temperatures and show good antibacterial activity. Therefore, this study aimed to identify thermophilic bacteria from the crater of Mount Tangkuban Perahu around West Java and assess antibacterial effectiveness of AMPs against *Streptococcus mutans*, which contribute to oral biofilm formation. The isolate obtained was identified using 16S ribosomal ribonucleic acid (rRNA) gene sequencing, and the supernatant of the isolate was tested against *S. mutans* American Type Culture Collection (ATCC) 25175 using the disc assay method. To determine AMPs-coding genes, its genome was uploaded to antibiotic and secondary metabolite analysis shell (antiSMASH) 5.0.0 platform and biofilm inhibition was tested using the microtiter plate technique (with a 96-well bottom). Subsequently, the results were assessed using a microplate reader operating at 595 nm wavelength. The isolate was identified as *Geobacillus subterraneus*, with antibacterial activity against *S. mutans*, and produced an inhibition zone of 8.40 mm at an optimum pH of 8. The output of AMPs-coding gene showed that AMPs of the isolate were a member of the lanthipeptide class I, or bacteriocin-I group. AMPs of *G. subterraneus* suppressed the growth of *S. mutans* biofilm at a supernatant concentration of 5%, with the lowest optical density (OD) value of 0.061 and the highest percentage of biofilm growth inhibition at 28.24%. Based on the results, *G. subterraneus* derived from the crater of Mount Tangkuban Perahu showed potent antibacterial properties against *S. mutans*, making it a promising novel *S. mutans* anti-biofilm candidate.

## 1. Introduction

Thermophilic bacteria, which thrive in high-temperature environments, are valuable biochemical sources capable of producing thermostable enzymes, antibiotics, antifungals, and anticancer drugs [1]. These bacteria produce secondary metabolites in the form of antimicrobial peptides (AMPs), which are stable at high temperatures and show effectiveness against gram-positive and gram-negative bacteria [2]. Therefore, this shows potential for exploring alternative antibiotics, particularly for bacterial resistance due to excessive and improper antibiotic use [3]. Studies have shown that thermophiles such as *Geobacillus* species isolated from the Jordanian hot springs of Zara produced AMPs or bacteriocins [4].

All living things have innate immunity, caused by low molecular weight proteins called AMPs. These proteins possess broad-spectrum antimicrobial and immunomodulatory activity against bacteria, viruses, fungi, and parasites [5]. Most AMPs have good activity against gram-negative and gram-positive bacteria, characterized by small (<10 kDa), cationic, and amphipathic molecules that typically include 6–50 amino acid residues [6, 7]. Microbial resistance to AMPs is considerably slower or delayed compared to conventional antibiotics. Therefore, AMPs have been suggested as a potential alternative therapy in the future. The mechanism of exerting antibacterial effect includes interfering with the biological operation of bacterial cell membranes by producing transmembrane pores, preventing bacterial survival. Another advantage includes rapidly eradicating antibiotic-resistant bacteria, including those resistant to methicillin and vancomycin [8].

To lessen the cariogenic qualities, *Streptococcus mutans* biofilm must be prevented from growing and becoming more virulent [9, 10]. Bacteria in biofilm show highly adaptive resistance to antibiotics and other disinfectants compared to the planktonic compartment, making treatment difficult. Biofilm has an extracellular polymeric substance (EPS) matrix, which serves as a physical barrier to protect the bacteria inside [7, 11]. Although conventional antibiotics commonly used to eliminate planktonic bacteria are applied to biofilm, it often results in ineffective treatment. This is because biofilm is more resistant to conventional antibiotic therapy, which reduces the efficiency of both treatment and eradication [12]. Consequently, numerous studies have been conducted to investigate novel strategies and AMPs as substitutes for treating biofilm infections.

Several natural AMPs have been shown to possess antimicrobial and anti-biofilm properties in vitro [13, 14]. However, these AMPs still have limitations, including a short half-life due to the susceptibility to protease degradation, inactivity at physiological salt concentrations, and potential cytotoxicity to host cells [13]. Biological fluids such as plasma, serum, and saliva can reduce the effectiveness of AMPs as anti-biofilm, leading to the formation of naturally generated AMPs with acquired stability [14, 15]. This necessitates studies to identify new ecological opportunities for bacteria capable of producing powerful, stable, and effective AMPs at low concentrations. Thermophilic bacteria are stable, proteolytically resistant, quickly lyse pathogenic microorganisms, and innocuous [16]. Therefore, this study aimed to identify thermophilic bacteria from hot springs in the crater of Mount Tangkuban Perahu and evaluate the activity of AMPs against *S. mutans*, contributing to anti-biofilm compounds.

## 2. Materials and Methods

### 2.1. Samples Isolation and Screening

A total of five water samples were collected from a hot spring in the crater on Mount Kamojang in Garut, and one was taken from the crater on Mount Tangkuban Perahu in Bandung, Indonesia, both at an elevation of 6837 and 5680 feet, respectively. During the sampling period, the pH of Mount Tangkuban Perahu was between 1.0 and 2.0 with a temperature of 45–76°C, while Mount Kamojang ranged from 63 to 95°C with a pH of 2.45–3.0. The samples obtained were placed in a test tube containing 100 mL of liquid thermus media and incubated at 55°C. The growth bacteria were then purified by subculturing on an Muller–Hinton agar (MHA) solid medium (Oxoid) under the same conditions. The isolates were screened by inoculating nutient broth (NB) media with bacteria at a concentration of 10% (v/v), and incubating the mixture for 24 h at 55°C and 150 rpm. The isolates were centrifuged using a tabletop centrifuge for 15 min at 10,000 rpm after 24 h of incubation to obtain cell-free supernatant. Subsequently, antibacterial activity of these purified isolates was tested against *S. mutans* American Type Culture Collection (ATCC) 25175 using a disc diffusion test.

### 2.2. Identification of Bacteria and 16S Ribosomal Ribonucleic Acid (rRNA) Gene Sequencing

According to Buchanan and Gibbons, the selected and purified isolate colonies were initially examined for size, pigmentation, form, margins, and elevation. Gram staining was then used to microscopically observe the colonies for characterization [17]. Subsequently, bacterial DNA was extracted for molecular microorganism identification using a genomic DNA purification kit (gDNA Presto Mini). 16S rRNA gene was amplified using the extracted DNA in a polymerase chain reaction (PCR) with the universal bacterial primers F27 (5 “AGAGTTTTGATCMTGGTCAG3” and R1492 (5 “TACGGYTACCTTGTTACGACTT3”). PCR products were compared using a 1 kb DNA ladder (Promega) before sequencing and examination by Genetika Science Indonesia. The results were submitted to National Center for Biotechnology Information (NCBI) [18, 19].

### 2.3. Production of Antimicrobial Compound

#### 2.3.1. Supernatant Preparation and Subculture of Test Bacterium

A fermentation experiment was conducted to obtain antibacterial metabolites from selected isolates. Approximately 20 mL of minimal medium liquid (MM; Solution 1 (g/L): K_2_HPO_4_: 0.23, KH_2_PO_4_: 0.5, NH_4_NO_3_: 0.3, and yeast extract: 0.5 and Solution 2 (g/L): nitrilotriacetic acid: 200, MgSO_4_.7H_2_O: 145.44, CaCl_2_.7H_2_O: 133.78, and FeSO_4_.7H_2_O: 11.12) was inoculated with 2.22 mL inoculum in a shaking incubator for 24 h at 55°C, 150 rpm, and optimum pH. After 24 h of incubation, the bacteria in the stationary phase were centrifuged for 20 min at 4°C and 10,000 rpm. To obtain a cell-free supernatant, the bacterial cell and supernatant were separated using a 0.22 µm Sartorius filter and stored at 4°C [20]. Subsequently, *S. mutans* ATCC 25175 from the microbiology lab, Faculty of Medicine, Universitas Padjadjaran, was used as test bacterium, subcultured on trypticase soya agar (TSA) and incubated at 37°C for 24 h [21].

#### 2.3.2. Antimicrobial Activity Assay

The production of antimicrobial metabolites was evaluated using a disc diffusion technique. The test bacteria were suspended in 9% NaCl solution, measured to achieve 0.5 McFarland standard solution, equivalent to 1.5 × 10^8^ CFU/mL, and swabbed on MHA agar media with 2% glucose. Subsequently, sterile disc paper was placed in a vial with *Geobacillus subterraneus* GE1 supernatant and allowed to soak for an hour at 4°C. The disc was placed on a petri dish with agar media that contained the test microorganisms. The ampicillin (Oxoid) disc was used as the positive control, while aquadest was used as the negative control. The petri dish was incubated at 37°C for 24 h, and the diameter of the inhibition zone formed was measured.

#### 2.3.3. Identification of the AMP Coding Gene

*G. subterraneus* genome sequences of the isolates were uploaded to the Bacteriocin Genome mining Tool A4 (BAGELA4) web server (http://bagel4.molgenrug.nl) to identify and characterize AMPs-coding gene for the bacteria. This process was followed by confirmation on antibiotic and secondary metabolite analysis shell (antiSMASH) 5.0.0 platform [22], and the coding gene sequences were analyzed and arranged using the NCBI database.

#### 2.3.4. Determination of Minimum Inhibitory Concentration (MIC)

The microdilution method was used to evaluate the antibacterial activity of peptides in 96-well polystyrene U-bottom plates. To each well, 100 μL Muller–Hinton Broth (MHB) containing supernatant at concentrations ranging from 20% to 2.5% was added, followed by 10 μL bacterial solutions containing 1.5 × 108 CFU/mL. In this study, chlorhexidine gluconate (0.12%) and MHB medium were used as positive and negative controls, and the plates were incubated for 24 h at 37°C. Using a microplate reader (Multiskansky), the absorbance of each well was assessed at 595 nm following incubation. MIC was the lowest peptide concentration capable of inhibiting bacterial growth [23].

#### 2.3.5. Biofilm Microplate Assay

Biofilm inhibition test was carried out according to da Silva et al. [23], using 96-well polystyrene "flat" bottom plates to determine the effects of *G. subterraneus* on biofilm formation. The plates were prepared in the same manner as MIC determination. After incubating for 24 h at 37°C, the plates were washed thrice with sterile distilled water. This was followed by adding of 200 μL of 99.8% methanol for 15 min to fix the adhered cell before emptying the plates and allowing to dry. After methanol removal, 200 μL of 1% crystal violet was added to each well for staining the biomass for 5 min. Subsequently, the plates were washed by running tap water and dried at room temperature. To dissolve the dye bound to biofilm mass, 200 μL of 33% acetic acid was added to the wells, and the absorbance was measured at 595 nm with a microplate reader. The percentage of biofilm inhibition was calculated using the following formula [20, 23]:

  $$\begin{array}{c} \%\text{ biofilm inhibition}=\frac{\text{Grow control OD−test sample OD}}{\text{Grow control OD}}\times 100. \end{array}$$

## 3. Results

### 3.1. Screening of Bacterial Isolates

A total of five isolates were collected from the Crater of Mount Kamojang, including GE2, GE3, GE4, GE5, and GE6, while GE1 was obtained at the crater of Mount Tangkuban Perahu. From the six purified isolates, antibacterial activity of GE1 showed the highest activity against *S. mutans*, generating a 7.74 mm zone of inhibition, as shown in Table 1. Consequently, GE isolate required further treatment.

### 3.2. Characterization of GE1

Isolate GE1 was successfully cultivated at pH 6 and 55°C, which were the optimum conditions as observed from the growth of bacterial colonies. Morphologically, GE1 is a motile, spore-forming, gram-positive rod, typically arranged in pairs, characterized by yellowish-white colonies that are circular and flat with whole edges. Based on molecular identification, the 16S rRNA target gene was successfully amplified by the 27F and 1429R primers by over 700 kb. Using a basic local alignment search tool (BLAST) search of the NCBI database, the acquired 16S rRNA gene sequences were compared to the variations in GenBank. Based on 16S rRNA genome analysis, isolates were 99.9% similar to *G. subterraneus* (GenBank accession no. CP051162.1), as shown in Table 2.

### 3.3. Antimicrobial Activity

**Figure 1:**
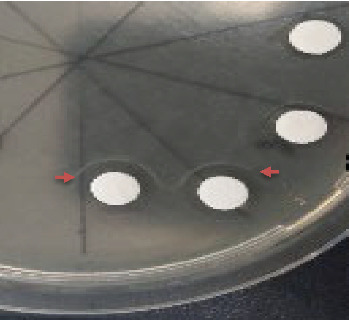
Antibacterial activity of the cell-free supernatant was obtained based on the growth phase. Colonies that grew in the initial stationary phase, at pH 8, were used to assess antibacterial activity. At this point, many bacteria produced the most antimicrobials. The test bacterium was *Streptococcus mutans* and the red arrow points to the inhibitory zone associated with antibacterial activity of the supernatant produced by *Geobacillus subterraneus*.

Based on the previously defined bacterial growth curve, the establishing phase started from the log, exponential, stationary, and death phases.  Table 3 shows the results of antimicrobial test. The isolates used to test antibacterial activity were obtained from an environment with a high bacterial population at pH 8 with intervals of 8–10 h. This environment was capable of producing a significant number of antibiotic compounds [24]. The largest inhibitory zone, measuring 8.40 mm, was produced by the early stationary phase of *G. subterraneus* used for antibacterial test against *S. mutans*, as shown in Figure 1.

### 3.4. *G. subterraneus* AMPs-Coding Gene

The results of the BAGELA4 study showed that the identified bacteria contained a sequence that was 100% similar to lantibiotic precursors (GenBank accession number BAD74579.1). Moreover, leader and core proteins constituted these peptides. After the leader peptide was removed from its amino acid sequence, the core peptide developed into a mature and functional lantipeptide [4]. AntiSMASH investigation discovered a group of genes that activated core peptides, specifically genes with sequence arrangement that were 100% identical to lantibiotic dehydratase (GenBank accession number WP 014194788.1) and lanthionine synthetase (GenBank accession number WP 020279663.1).

### 3.5. Biofilm Microplate Assay

**Figure 2:**
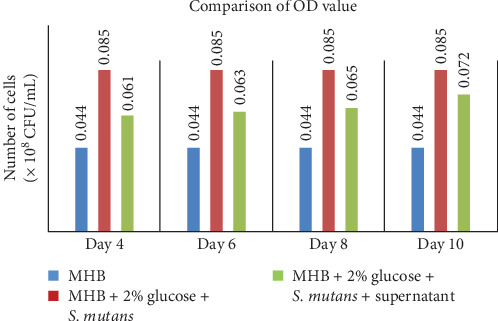
Comparison of OD measurements between negative control and treatment control. Inhibition of *Streptococcus mutans* biofilm formation by supernatant of *Geobacillus subterraneus* was assayed on polystyrene microtiter plates and stained with crystal violet. A total of four experiments were performed on days 4, 6, 8, and 10. Negative control (blue), growth control (red), and biofilm inhibition tested (green) were also tested. OD, optical density; *S*. *mutans*, *Streptococcus mutans*.

In this study, an anti-biofilm test was carried out at intervals of 4–10 days of incubation. Antibacterial activity of *G. subterraneus* supernatant peaked on day 4 with an optical density (OD) value of 0.061 and gradually decreased. The growth of *S. mutans* was restricted by the supernatant of *G. subterraneus*, which also affected biofilm development.  Table 4 shows that the growth of *S. mutans* biofilm is significantly decreased. The efficiency of inhibiting the growth of biofilm was assessed by comparing OD negative control (MHB), growth control (MHB, 2% glucose, and *S. mutans*), and treatment (MHB + 2% glucose + *S. mutans* + supernatant; Figure 2). Based on this comparison, AMP produced by *G. subterraneus* showed the capacity to dissolve *S. mutans* biofilm beginning on day 4 with the lowest OD value and the highest biofilm inhibition percentage of 28.24%. As shown in Table 5, antimicrobial efficacy started to diminish the following day and did not fully recover until day 10, with the highest OD value and the lowest biofilm inhibition percentage of 14.82%.

## 4. Discussion

Previous studies showed that AMPs were capable of inhibiting planktonic bacteria and biofilm-related oral health [9]. Biofilm is a bacterial community where cell-to-cell communication occurs (Quorum sensing), attaching to multicellular surfaces, and is embedded in the extracellular matrix. Bacteria living in biofilm show a significantly higher pattern of adaptive resistance to antibiotics and other disinfectants compared to the planktonic compartment, making treatment difficult [11]. Consequently, several studies have been conducted to obtain a substitute for antibiotics that can inhibit the growth and development of oral biofilm to control pathogenic oral microflora. Several AMPs were also clinically tested for dental and oral diseases, showing antimicrobial effects and good anti-biofilm activity [11]. This study is the first to investigate the activity of AMPs as *S. mutans* anti-biofilm. Based on the results, AMP of GE1 inhibited planktonic *S. mutans* and single-species *S. mutans* biofilm.

GE1 was identified as *G. subterraneus*, a gram-positive bacterium that thrives in aerobic or facultative anaerobic conditions. *Geobacillus sp*. is commonly found in environments with high temperatures ranging from 30 to 80°C [24]. *G. subterraneus* GE1, a thermophilic bacterium, is capable of producing AMP at 55°C, with moderate antibacterial activity against *S. mutans* at an optimum pH of 8. Thermophilic bacteria from various sources have also been found to produce AMPs that are active against gram-positive and gram-negative bacteria. However, the results varied slightly from a previous study on *G. toebii* HBB-247, a thermophilic bacterium isolated from hot springs, which produced stable bacteriocins at 60°C [17]. According to antiSMASH analysis, *G. subterraneus* GE1 produced a class 1 lanthipeptide belonging to the bacteriocin-I group, which are efficient AMP for gram-positive bacteria [25]. Similarly, Egan et al. [26] carried out an in-silico screening analysis using BAGEL 3, where the coding gene and lanthipeptides were retrieved. This shows that *Geobacillus sp*. is an effective AMPs generator.

Compared to mesophilic bacteria, *Geobacillus sp*. is an excellent AMPs producer and a potential source of stable proteins for harsh environments with high temperatures [24]. Bacteriocins-I are proteolytic, heat resistant, and characterized by low molecular weights of less than 5 kDa. These peptides comprise amino acids such as lanthionine and methyllanthionine and pass through posttranslational modification and cleavage for nontoxic or mildly toxic bacteriocin to mature. One of these groups is the lantibiotics, which are lanthipeptides that show potent antibacterial activity against gram-positive bacteria by forming pores in the bacterial cell membrane [27]. The amphiphilic cationic nature of these peptides results in negatively charged pores in the bacterial cell membrane. This phenomenon allows the small metabolites of the susceptible bacterial cells to drain out and causing death [28]. AMPs prevent biofilm formation by interfering with initial adhesion, cell-to-cell communication, the formation of biofilm matrix, and the capacity to dissolve biofilm [12]. These results correspond with previous studies showing that gram-positive bacteria, specifically *G. subterraneus*, produce class I lanthipeptide AMP. Furthermore, this AMP show antibacterial properties against gram-positive bacteria, particularly *S. mutans* [29].

In this study, the cell-free supernatant was collected during the transition from the logarithmic to the stationary growth phase. Generally, lanthipeptides or bacteriocins are produced by dormant ribosomes and released during the bacterial logarithmic growth phase. This process produced the supernatant after the logarithmic stage and before the stationary phase [30]. To obtain maximum antibacterial compounds, temperature, and pH are critical factors. In this study, the maximum supernatant was obtained at a temperature of 55°C with an incubation time of 24 h, with the best activity found at 48 h, which was consistent with the results of Muhammad and Ahmed [17]. pH also played a significant role in the production of antibacterial compounds. For instance, *G. subterraneus* GE1 grows most quickly at a pH of 8, where the highest levels of antibacterial compound synthesis occur. pH of the fermentation medium affects various cellular processes, including the regulation and control of microbial bioactive metabolite biosynthesis [17]. The microtiter-plate method was used to identify and quantify the growth of *S. mutans* biofilm. Additionally, MHB media with 2% glucose was used to enhance biofilm formation by upregulating the expression of the gene responsible for producing polysaccharide intracellular adhesion (PIA) [25]. PIA is a biofilm component that assists in adhering planktonic bacterial cells during maturation. Bai et al. [25] also added 1% glucose to the media to induce *S. aureus* biofilm production.

According to the scientific literature, oral bacterial biofilm has not been tested against AMPs generated from thermophilic bacteria [31]. This study evaluated novel features of antibacterial chemicals that thermophilic bacteria create as anti-biofilm. *S. mutans*, a gram-positive oral bacterium, the predominant microorganism during the early phases of biofilm development, was susceptible to the antibacterial action of AMP generated by *G. subterraneus* GE1. Furthermore, an anti-biofilm test of *G. subterraneus* discovered that *S. mutans* biofilm could be prevented at a supernatant concentration of 5%, which is low and presumably incapable of impacting the normal microflora of the oral cavity [31]. This study focused on how the single-species biofilm bacterium *S. mutans* was affected by AMP of *G. subterraneus*. Previous investigation showed that removing *S. mutans* significantly lowers its volume, limiting biofilm formation in the oral cavity. The inhibition of *S. mutans* will also reduce biofilm growth and aid in averting several oral health issues [22]. AMPs limit the development of biofilm by interfering with initial adhesion, cell-to-cell communication, the production of biofilm matrix, and dissolution ability [12]. This study shows that *G. subterraneus* AMP have the potential as anti-biofilm rather than inhibitors of *S. mutans* growth. According to current knowledge, anti-biofilm is a natural or produced process that reduces bacterial biomass by changing biofilms' development, integrity, and quality. Meanwhile, the term “inhibitor” describes the prevention of bacterial surface attachment and the instability or rupture of mature biofilms that have become permanently adherent. In a previous study, the growth of *S. mutans* can be inhibited by preventing bacterial attachment to surfaces and destabilizing or irreversibly destroying the attachment of mature biofilm [32].

Based on the definition, *G. subterraneus* AMP has anti-biofilm properties that change the growth and lower bacterial biomass. AMPs are potential solutions for preventing oral biofilm growth due to their safety, lack of staining or irritation of teeth, and lack of odor, color, and taste. Furthermore, this peptide prevent biofilm from adhering to the tooth surface [26]. Meanwhile, *S. mutans* biofilm is immune to antibiotics, creating an extracellular matrix barrier and biofilm enzymes that are physiologically resistant to traditional antimicrobials and well-adapted to the oral environment [22]. Based on the results, thermophilic AMP from *G. subterraneus* bacteria is a more reliable and effective anti-biofilm option for *S. mutans* in the oral cavity. The results of this study are preliminary and can be applied to further preclinical and clinical studies.

## 5. Conclusion

In conclusion, *G. subterraneus*, identified from the hot springs of Tangkuban Perahu, can produce AMP, a class I lanthipeptide belonging to the bacteriocin I family. The AMP possessed antibacterial activity against gram-positive bacteria *S. mutans*. Based on the results, *G. Subteraneus* AMP showed potential as a candidate for the prevention and prophylaxis of *S. mutans* biofilm. This *S. mutans* anti-biofilm test also proved that the AMPs could prevent biofilm formation at low concentrations.

## Figures and Tables

**Table 1:** Antibacterial activity of thermophilic bacterial isolates against *Streptococcus mutans*.

Indicator bacterial	Inhibition zones formed by thermophilic bacterial isolates (mm)					
*Streptococcus mutans*	GE1	GE2	GE3	GE4	GE5	GE6
	7.74	6.49	7.26	6.38	7.02	7.56

**Table 2:** Characterization of bacterial isolate GE1.

Analysis	Examination	Characteristic	Result
Morphological analysis	Microscopic	Gram staining	+
		Shape	Rod
		Spore	+
		Motility	+
	Macroscopic	Color	Yellowish-white
		Elevation	Flat
		Colony shape	Circular
		Margin	Entire

Molecular analysis	Universal primer 27F and 1429R	—	99.9% similarity to *Geobacillus subterraneus* (GenBank accession no. CP051162.1)

**Table 3:** Antimicrobial activity of *Geobacillus Subterraneus* GE1 against *Streptococcus mutans*.

Isolate	Growth phase	Zone of inhibition (mm)
*Geobacillus subterraneus* GE1	End log	7.35
	Initial stationary	8.40
	Stationary	7.74
	Final stationary	6.89

K+	—	20.82

K−	—	0

Abbreviation: K, control.

**Table 4:** Results of OD measurement (595 nm).

Treatment						
MIC	Day 4	Day 6	Day 8	Day 10	MHB + SM	MHB
5%	0.061	0.063	0.065	0.072	0.085	0.044

Abbreviations: MHB, Muller–Hinton Broth; MIC, minimum inhibitory concentration; SM, *Streptococcus mutans* ATCC 25,175.

**Table 5:** Biofilm inhibition percentage.

Days of incubation	Biofilm inhibition (%)
Incubation day 4	28.24
Incubation day 6	25.88
Incubation day 8	23.53
Incubation day 10	14.8
Average	23.12

## Data Availability

The author provides the data used or analyzed during the current study upon reasonable request.
